# Lower Body Positive Pressure Application with an Antigravity Suit in Acute Carotid Occlusion

**DOI:** 10.4061/2010/950524

**Published:** 2010-04-01

**Authors:** Karine Berthet, Anne Claire Lukaszewicz, Marie-Germaine Bousser, Didier Payen

**Affiliations:** ^1^Department of Neurology, Lariboisière Hospital, Assistance Publique Hôpitaux de Paris and University Paris 7 Diderot, 2 rue Ambroise Paré, 75475 Paris Cedex 10, France; ^2^Department of Anesthesiology & Critical Care Medicine, Lariboisière Hospital, Assistance Publique Hôpitaux de Paris and University Paris 7 Diderot, 2 rue Ambroise Paré, 75475 Paris Cedex 10, France

## Abstract

The challenge in acute stroke is still to reperfuse as early as possible the ischemic territory. Since fibrinolytic therapies have a limited window with potential risk of bleeding, having a nonpharmacologic mean to recruit vessels in area surrounding necrosis might be useful. We propose here to use antigravity suit inflated at “venous” pressure levels to shift blood towards thoracic and brain territories. We report two cases of spectacular clinical recovery after acute carotid occlusion.

## 1. Introduction

We report two cases where an anti-gravity suit (also named MAST: Medical Antishock Trousers [1, 2]) was applied with a low gradient of pressure during the acute phase of symptomatic carotid occlusion to amplify the blood volume shift towards the craniothoracic territory [3, 4], improving cerebral haemodynamic conditions and neurological symptoms. The impact of anti-gravity suit application on cerebral vascularisation was measured by transcranial Doppler.

## 2. Case  1

A 28-year-old woman was seen 2 hours after the sudden onset of total right brachial monoplegia, right facial palsy, and mutism (NIH Stroke Scale: 17) due to a left middle cerebral artery (MCA) infarct. She received intravenous recombinant tissue plasminogen activator (rt-PA) 2.5 hoursafter stroke onset, without subsequent improvement. Cerebral MRI diffusion-weighted images and T2 flair-weighted images performed 24 hours later showed increased signal in the whole superficial left MCA territory (Figures 2(a) and 3(a)). The MR Angiography (MRA) showed a proximal left internal carotid artery (ICA) occlusion, a tight stenosis on the distal right ICA, no signal in the right siphon and right MCA, while there was a weak signal in the left MCA (Figure 4). T1-FAT-SAT-weighted images showed bilateral ICA dissection (Figure 5). Doppler study showed a high resistance to flow in both common carotid arteries and low bilateral MCA flow with low systolic and diastolic velocities. Intravenous heparin treatment was started 24 hours after rt-PA administration and the patient was lying in a strict −20 degrees head down tilt [5].

Two days after stroke onset, there was no clinical improvement and the patient developed bradycardia (40 pulses/min) and hypotension (100/70 mmHg). Lower body positive pressure (LBPP) was applied using an anti-gravity suit (Trauma Air Pants; Life Support Products Inc., St Louis, Missouri, USA) for 150 minutes. The anti-gravity suit included three independent bladders connected to three pneumatic extremities with manometers providing instantaneous pressure values. The garment was applied from ankle to costal margin and the bladder pressures were set at 20 mmHg on the lower limbs and 10 mmHg on the abdominal area, to keep a positive pressure gradient [3, 4]. During the procedure the patient was lying in a supine position. The following parameters were recorded at baseline, after intravenous infusion of 500 mL of fluid load (colloid), 15 minutes and 150 minutes during LBPP application and 10 minutes after gravity suit deflation: right arm mobility, blood pressure, heart rate, systolic and diastolic velocities recorded on both MCAs and resistance index (RI) recorded on both common carotids arteries (CCAs).

**Figure 1:**
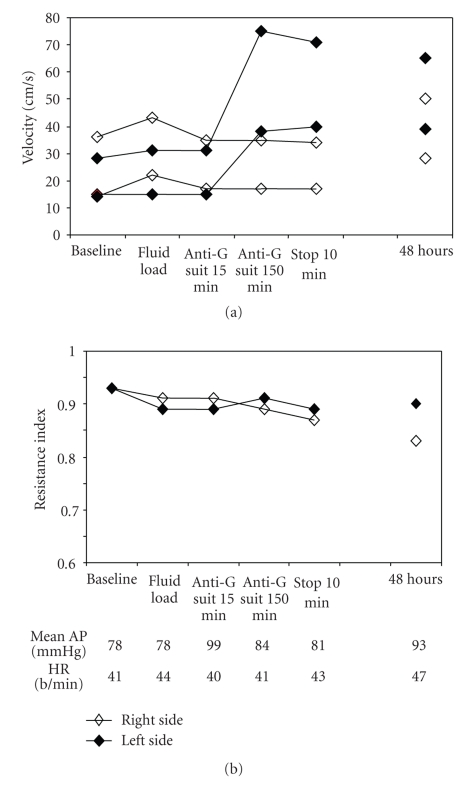
(a) Patterns of intracranial MCA blood flow velocities with time in both the right and left sides, before, during, and after (10 minutes and 48 hours) LBPP application. Note the left side improvement in systolic and diastolic blood flow velocity induced by LBPP, which was sustained for at least 2 days. (b) Vascular resistance index (RI) evolution (method for computation: systolic V/diastolic V). A clear difference between right and left RI occurred only after 48 hours of LBPP application. Corresponding mean arterial pressure (mean AP) and heart rate (HR) at each time.

During LBPP application and up to 48 hours later, there was a 50% increase in systolic and diastolic MCA velocities (Figure 1). The RI decreased on both CCAs from 0.93 to 0.86 and continued to decrease on the right side after the procedure. Heart rate and blood pressure remained stable during LBPP application and during the proceeding days.

Three days after LBPP application the patient started to recover her right hand mobility and her facial palsy started to improve. One week after LBPP application she had completely recovered her right arm and facial mobility. She finally went back home 3 weeks after hospitalisation.

**Figure 2:**
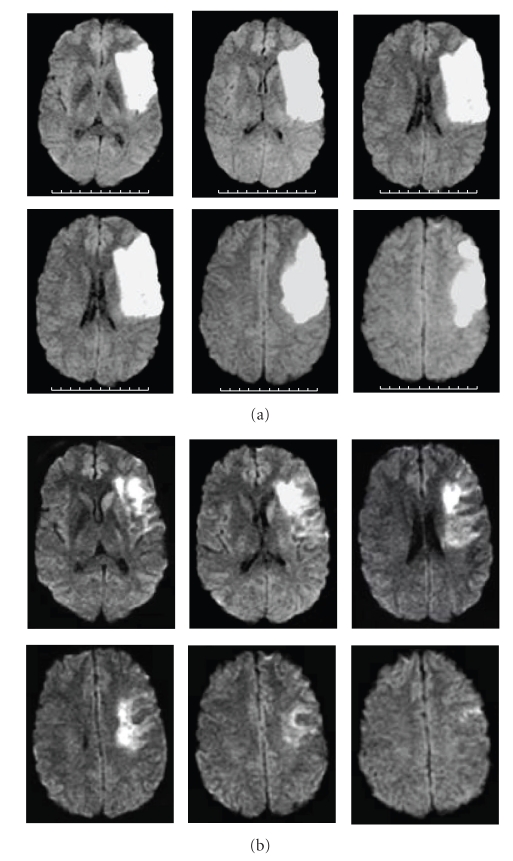
MRI diffusion-weighted images showing left superficial MCA infarct. (a) 24 hours after stroke onset, (b) 5 days after stroke onset.

**Figure 3:**
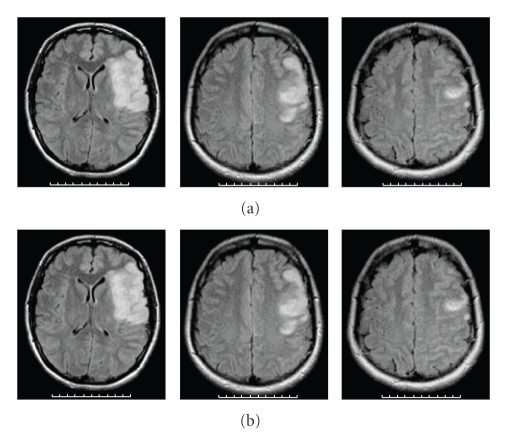
MRI T2 flair-weighted images showing left superficial MCA infarct. (a) 24 hours after stroke onset, (b) 5 days after stroke onset.

**Figure 4:**
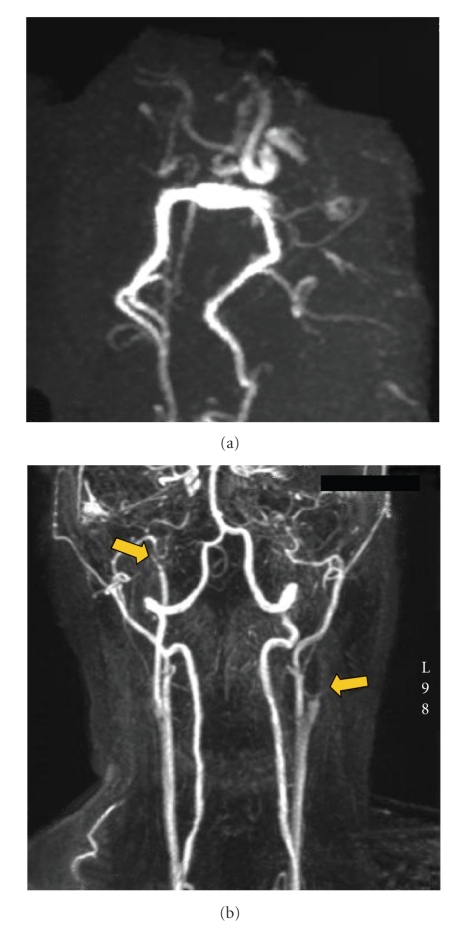
(a) Intracranial MRA of Willis circle: right siphon, right and left MCAs were not visible. Posterior communicating arteries were also not visible, with visible posterior cerebral arteries. (b) Cervical MRA showing proximal left internal carotid artery occlusion and distal right internal carotid artery tight stenosis (arrows).

**Figure 5:**
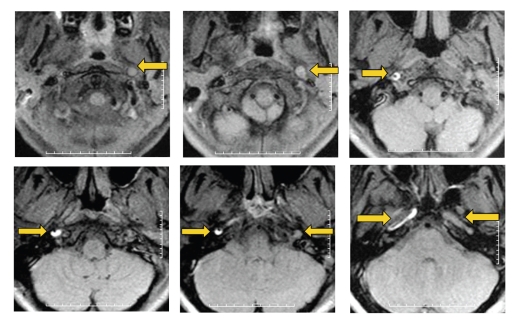
MRI T1-FAT-SAT-weighted images showing the dissecting process visible as a hypersignal in the wall of both ICAs as well as an enhancement of ICA diameter (arrows).

**Figure 6:**
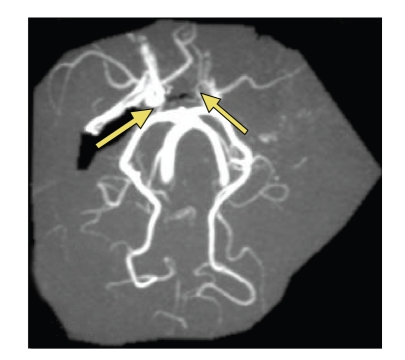
Intracranial MRA showing recanalisation of the right ICA; the right and left MCAs are visible as well as both posterior communicating arteries (arrows).

On MRI performed 5 days after stroke onset, diffusion-weighted images showed reduced diffusion abnormalities and reduced infarct size on T2 flair-weighted images (Figures 2(b) and 3(b)). The MRA showed recanalisation of the right ICA with good visualisation of siphon and MCA but a persistent left ICA occlusion (Figure 6).

## 3. Case  2

A 61-year-old. man was admitted in our stroke unit after 2 reversible episodes of left monocular blindness associated with a total right distal upper limb paralysis. His medical past history was hypertension, hypercholesterolemia, and tobacco use. At the admission the clinical examination showed a total right wrist extension paresis, the so-called “pseudoradial paresis” related to a small left frontal infarction visible on MRI (DWI). The MR angiography (MRA) and the duplex scanning showed an occlusion of the left internal carotid artery due to atherosclerosis and a negative flow in the ophthalmic artery downstream. The intracranial MRA showed poor collateral supply by anterior communicating artery and an apparent nonfunctional posterior communicating artery. We decided to apply LBPP process for 2 reasons: (1) the worsening of the right hand deficit (see movie), (2) this worsening was not related to a fall in systemic blood pressure (167/84 mmHg). The LBPP was applied once during 90 minutes following the same procedure than in case one. The repeated measurements of blood pressure and heart rate did not show any significant changes along the procedure. The transcranial Doppler showed a decreased of the left MCA resistance to flow index ([systolic velocity-diastolic velocity]/systolic velocity, 0.5 at baseline to 0.4 at the end of the procedure) while there was slight changes on the MCA velocities (64/32 to 75/45 cm/sec). The left ophthalmic artery flow remained negative during and after LBPP application. Heart rate and blood pressure remained stable during LBPP application. The clinical improvement was sustained after anti-G suit deflation (as presented on the movie) consisting in the recovery of the pseudoradial palsy with persisting ataxia lasting few days. The total recovery was obtained before discharge.

## 4. Discussion

We report two patients with symptomatic carotid artery occlusion in whom LBPP was applied with an anti-gravity suit during the acute phase of stroke. The patients made a remarkable recovery in parallel with significant cerebral hemodynamic improvement not related to global systemic hemodynamic changes.

LBPP is known to shift blood from the lower body compartment towards the upper part of the body, which improves cardiac preload and output [4, 6, 7] and potentially cerebral perfusion [8]. The addition of an abdominal compression to lower limbs was supposed to be more efficient to push more blood to the brain than compression of lower limb only [6].

 Several mechanisms might be discussed for neurological improvement: (1) recanalization of carotid occlusion, (2) development of collaterals, and (3) recruitment of smaller vessels in watershed fields. For the first patient, it is unlikely that rt-PA administration played a major role in this dramatic improvement since there was no improvement 24 hours after treatment, which decreases the likelihood of a favourable outcome to 20% [9, 10], and the efficacy of rt-PA in carotid occlusions is debatable [11, 12]. 

The reopening of the carotid artery, which was unusually rapid for the spontaneous evolution of a dissection [13], may have played an important role in case 1 evolution. Whether LBPP application contributed to this reopening, though likely with RI decrease on right CCA, is open to debate and we cannot rule out coincidental recanalization of the ICA occlusion. In case 1, the parallel improvement in the clinical status and flow parameters on transcranial Doppler (TCD) during and after LBPP application suggests a beneficial effect of LBPP application on brain perfusion, particularly through restitution of blood flow in the ischemic left MCA territory. It could be due to the recruitment of collateral pathway through Willis circle explaining the increase of systolic and diastolic velocities recorded on left MCA during LBPP application (case 1, Figure 1), as well as the improvement of anterior cerebral circulation shown by the MRA performed 5 days after stroke onset (right siphon and right MCA were visible and the posterior communicating arteries were functional on Figure 6). The second case reported which showed a clinical improvement (film) during the LBPP application showed a weak improvement in cerebral velocities. The temporal relationship between clinical recovery and LBPP application suggests a benefit. 

Another possible beneficial effect could be the recruitment of smaller vessels in watershed fields especially in case 2. The clinical improvement during the LBPP application could be related to the new partition in the frontal region of blood which could play a role in the recruitment of small vessels by an efficient loading or even a reopening. 

Two recent reports showed the impact of a mechanical external counter-pulsation on cerebral velocities in healthy volunteers [8] and on functional outcome in patients with stroke [14]. In this study the LBPP application was limited to the lower limbs by the mean of a cyclic external counter-pulsation at diastolic time of heart cycle which is more complex than our classical anti-gravity suit. Furthermore, we can expect that the anti-gravity suit could mobilised a higher blood volume in respect of the abdominal compartment implication. 

Even if this paper is limited to two patients, without control group, the remarkable recovery observed in these two patients together with the improvement in hemodynamic parameters on TCD might be related to LBPP application through improvement of cerebral perfusion. We need definitively, further investigation during the acute phase of ischemic stroke to conclude on the beneficial effect of this noninvasive tool.
